# Adolescent utilization of sexual and reproductive health services in Gamo Zone, Southern Ethiopia. Insights from multilevel and latent class analysis

**DOI:** 10.3389/frph.2024.1356969

**Published:** 2024-07-03

**Authors:** Negussie Boti Sidamo, Amene Abebe Kerbo, Kassa Daka Gidebo, Yohannes Dibaba Wado

**Affiliations:** ^1^ School of Public Health, College of Health Sciences and Medicine, Wolaita Sodo University, Wolaita Sodo, Ethiopia; ^2^ School of Public Health, College of Medicine and Health Sciences, Arba Minch University, Arba Minch, Ethiopia; ^3^ African Populations and Health Research Center, Nairobi, Kenya

**Keywords:** adolescent health, sexual and reproductive health services, utilization, multilevel analysis, latent class analysis, health services access, Gamo Zone, Southern Ethiopia

## Abstract

**Introduction:**

Adolescents face unique challenges related to their sexual and reproductive health (SRH), with access to timely services being critical for positive outcomes. However, SRH service utilization among adolescents remains low. This study aimed to identify factors influencing SRH service use among adolescents in Gamo Zone, Ethiopia.

**Methods:**

A community-based cross-sectional study involved 1172 adolescents selected through stratified sampling. Structured face-to-face interviews were employed for data collection. Multilevel mixed logistic regression was fitted to identify factors and latent class analysis was conducted to understand population heterogeneity.

**Results:**

The findings of this study reveal that 198 (16.89%) adolescents (95% CI: 14.8%-19.2%) utilized SRH services within the past 12 months. Factors significantly associated with SRH service utilization included good knowledge about SRH rights (AOR = 4.65; 95% CI: 2.68, 8.07), belonging to one-parent families (AOR = 4.13; 95% CI: 2.39, 7.12), engaging in parental discussions regarding SRH issues (AOR = 3.17; 95% CI: 1.89, 5.29), high family support (AOR = 1.96; 95% CI: 1.09, 3.51), and enrolling in school (AOR = 0.19; 95% CI: 0.11, 0.33). Additionally, access to social media was associated with increased SRH service utilization among adolescents (AOR = 1.98; 95% CI: 1.25, 3.15). Latent class analysis identified four groups: rural school-enrolled adolescents living with parents, urban school-enrolled adolescents with both parents, urban disadvantaged female adolescents, and early adolescents with limited social media access.

**Conclusions:**

In conclusion, our study sheds light on the utilization of SRH services among adolescents, revealing that 16.89% of the participants accessed these services within the past year. Significant factors associated with SRH service utilization included good knowledge about SRH rights, belonging to one-parent families, engaging in parental discussions regarding SRH issues, high family support, and enrollment in school. Interestingly, access to social media was also linked to increased utilization of SRH services among adolescents. Furthermore, our latent class analysis identified four distinct classes of adolescents based on socio-demographic indicators, highlighting the heterogeneity within this population. These findings underscore the importance of tailored interventions and targeted approaches to address the diverse needs of adolescents in accessing and utilizing SRH services.

## Introduction

Adolescent sexual and reproductive health (SRH) remains a critical area of concern globally, particularly in low- and middle-income countries where access to comprehensive SRH services is limited (1, 2). Each year, approximately 12 million adolescent girls aged 15–19 years give birth (2). Many adolescents engage in risky behaviors such as substance use, violence, and unsafe sexual practices, which heighten the risk of sexually transmitted infections (STIs), including HIV/AIDS (3–6). For instance, UNICEF reported that 160,000 adolescents worldwide were newly infected with HIV in 2021. Timely access to SRH services is crucial for adolescents health and wellbeing (7).

SRH services should be acceptable, accessible, appropriate, equitable, and effective for adolescents (8). The Guttmacher-Lancet Commission identifies key components of SRH services, including maternal and newborn care, contraceptive services, STI prevention and treatment, safe abortion services, sexual well-being counseling and support, gender-based violence prevention and counseling, and treatment for infertility and cervical cancer (9). The Nairobi Summit (ICPD + 25) emphasized the importance of these essential services in ensuring SRH choices and rights for all (10).

Despite global and national efforts, the utilization of SRH services among adolescents remaines lows (8, 11–15). In Ethiopia, like many low- and middle-income countries, SRH service utilization remains low, ranging from 8.6% to 39.5% (11, 16–20), despite the government's commitment to improving adolescent health and aligning with the Nairobi Summit's goals to ensure universal access to SRH services (21, 22).

Understanding the factors influencing adolescents' utilization of SRH services is essential for developing effective interventions and policies tailored to their needs (7, 23, 24). While previous studies on SRH service utilization among adolescents have primarily focused on individual-level factors such as age, gender, place of residence, sexual history, and SRH knowledge (11, 14, 25–27). These studies often overlook the specific contexts in which adolescents live, including family environment (family structure, support, parental monitoring, and discussions about SRH) and community-level factors like access to social media.

Moreover, existing research on adolescent SRH has predominantly focused on specific subsets of adolescents such as those residing in urban areas or enrolled in school, as well as female adolescents (11, 13, 18, 19, 28, 29). Additionally, many prior studies have tended to analyze individual components of SRH services in isolation, rather than adopting a comprehensive and integrated approach (13, 29). While these studies have provided valuable insights into particular demographic segments or services, they often fail to capture the diverse experiences and needs of all adolescents. This gap in the literature is concerning as it hinders the development of holistic and inclusive health interventions and policies that do not inadvertently exclude any group. A holistic approach can provide a broad overview of adolescent health and wellbeing, which is essential for comprehensive policy planning. Failure to address the varied needs of adolescents from different backgrounds not only perpetuates health disparities but also limits the effectiveness of interventions aimed at promoting adolescent well-being and preventing health issues later in life. To address this gap, comprehensive research considering diverse family and community-level factors is needed, as these significantly impact adolescents' behaviors and decisions regarding their SRH (3, 30–33). Moreover, there is a critical need for studies that encompass the full spectrum of adolescent experiences to inform more inclusive and effective public health strategies.

Therefore, this study employs a multilevel mixed effects analysis to investigate the utilization of SRH services among adolescents in the Gamo Zone, South Regional State of Ethiopia. By examining both individual and contextual factors, including family and community-level influences, this study aims to provide a comprehensive understanding of the determinants of SRH services utilization among adolescents. The findings of this study will contribute valuable insights to the existing literature on adolescent SRH in Ethiopia, informing the development of targeted interventions and policies to improve access to and utilization of SRH services. Ultimately, addressing the unique needs of adolescents in the Gamo Zone and similar regions is crucial for promoting their health and well-being and achieving broader public health objectives.

### Research question

This study is designed to address the following key research questions:

1. What is the magnitude of adolescent utilization of SRH services in the Gamo Zone?
2. What are the multilevel factors at the individual, household, and community levels that significantly influence the utilization of SRH services among adolescents in the Gamo Zone?
3. What are the distinct subgroups or patterns of sexual and reproductive health services utilization among adolescents?

## Materials and methods

### Study design and period

A community-based cross-sectional study was undertaken from March 2 to April 9, 2023.

### Study setting

This study was conducted in *the Gamo Zone*, one of the zones in the Southern Regional State of Ethiopia. Administratively Ethiopia is divided into 4 levels: the first level (*regions*), the second (*zones*), the third (*woredas*), and *kebeles* (the lowest administrative level) (34). *Each region* is divided into *zones*. Each *zone* is then divided into *woredas* (districts), and each district is divided into *kebeles* (the smallest administrative unit with 3,000–5,000 inhabitants). The *Gamo zone* borders the Wolayta and Gofa zones to the north, Lake Abaya to the northeast, the Amaro and Dirashe special woreda to the southeast, and the *South Omo* zone to the southwest. Arba Minch town is the administrative center of this Zone. This town is located 431 km from the Ethiopian capital city (Addis Ababa). Six town administrations and 14 rural districts with 306 *kebeles* were found in the Gamo zone. The total population in this zone is 1,643,205 of those 805,205 are male and 838,034 are female (35). There are currently 363 public health facilities providing preventive and curative services to the community. Of these, five are primary hospitals, one general hospital, 59 health centers, and 297 health posts. In addition, there are 251 private healthcare facilities. Of these, one primary hospital, 190 private clinics, 56 private pharmacies, and four drug stores. According to the 2023 performance report of the Health Department of Gamo Zone, family planning coverage was 78% of those 6.09% were adolescents aged 10–19 years. HIV testing coverage was 75%, ANC coverage 98%, and institutional delivery coverage was 73% (35).

### Sources and study population

The source population comprises adolescents residing in the Gamo Zone, ranging from ages 10 to 19 years old. The study population comprised all randomly selected adolescents within the selected study area during the selected study period who met the inclusion criteria.

### Inclusion and exclusion criteria

#### Inclusion criteria

The inclusion criteria for this study comprised adolescents aged 10–19 years residing in the Gamo Zone, South Regional State of Ethiopia. To ensure a comprehensive representation of adolescent experiences, individuals from both urban and rural areas within the Gamo Zone were included. Additionally, both male and female adolescents were encompassed in the study to account for gender diversity. This approach aimed to capture the diverse socio-economic and environmental contexts that adolescents navigate within the specified geographic area, thereby providing a holistic understanding of adolescent experiences related to the study's objectives.

#### Exclusion criteria

Participants who do not provide informed consent to participate in the study. Those adolescents with known hearing or mental impairments and/or those who were seriously ill at the time of data collection were excluded from the study. This exclusion criterion was implemented to maintain the integrity of the data collected and to focus on adolescents who could reliably participate in the study procedures.

### Sample size determination

To determine the sample size required for this study, Open Epi software version 3.01 was employed under the following assumptions: a confidence level of 95%, an absolute precision of 4% (*d* = 0.04), a power of 80%, and a proportion of adolescent SRH service use of 33.8%, as indicated by a previous study in central Ethiopia (21). The calculated sample size was 537. However, due to the utilization of a stratified sampling technique, it was necessary to adjust the sample size to accommodate for the design effect to address sampling variance and within-group differences among adolescents (36, 37). Thus, a design effect of two was applied, resulting in the multiplication of the primary sample size by two, yielding a required sample size of 1,074 participants. Considering a non-response rate of 10% due to the sensitive nature of the questionnaires, the final sample size was adjusted accordingly, resulting in a total of 1,181 participants.

### Sampling technique

**Figure 1:**
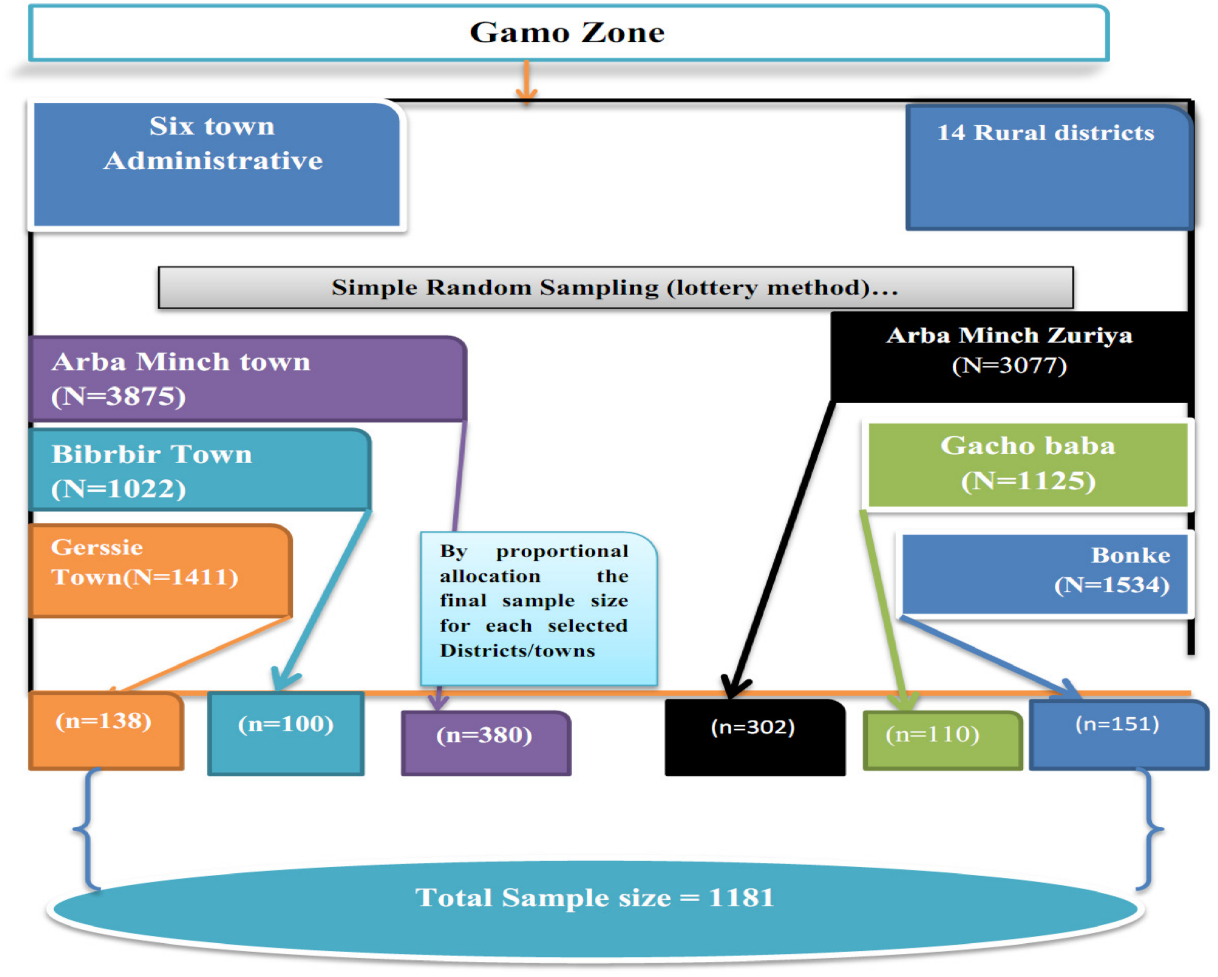
Diagrammatic presentation of sampling technique for the study on adolescent sexual and reproductive health services utilization in Gamo Zone, South Regional State of Ethiopia, 2023.

A multistage stratified sampling technique was used to select a representative sample of adolescents. In the first stage, the Gamo zone was stratified into urban and rural administrative strata. To ensure the precision of the survey in each stratum (urban vs. rural), an equal number of samples (primary sampling unit) were selected. This approach is commonly used in demographic surveys in Ethiopia (38). The use of implicit stratification and proportional allocation was achieved at each lower administrative level (*Kebele*) by sorting the sampling frame within each sampling stratum before sample selection (38). Three of the fourteen rural districts and three of the six town administrations were selected by lottery. In the second stage, 36 *kebeles* (11 urban *kebeles* and 25 rural *kebeles*) were selected with probability proportional to the *kebeles* in each stratum and with independent selection from each sampling stratum. Households with eligible participants (adolescents) were the third-stage sampling units sampled from the selected *kebeles*. Then the sample size was proportionally distributed among each of the selected *kebeles*. With the assistance of a health extension worker, sampling frames were created for each selected *kebele* using the family logbook. Then households who had eligible adolescents were selected using a simple random sampling technique. Finally, adolescents (10–19 years old) who were present in the household at the time of the visit and agreed to participate were interviewed. If there is, more than one eligible adolescent in the selected household, one was selected by lottery method (Figure 1).

### Variables of study

#### Dependent variable

Sexual and reproductive health services utilization among adolescents.

#### Independent variables

Individual, family/household and community-level factors were considered as independent variables in this study.

☛ **The individual-level** variables included in the study were age (10–14, 15–19), schooling status (enrolled and not enrolled), and knowledge about SRH right.
☛ **The family/ household level** variables included in the study were family structure (two-parent families, one-parent families, and Neither-parent families), adolescent-to-parental discussions regarding SRH issues (Yes, No), perceived parental monitoring, and family support.
☛ **Community-level** variables included in the study were residence (urban, rural), perceived social norm (approval or disapproval of receiving SRH services), and exposure to social media.
☛ All these variables were considered based on their theoretical and practical relevance to SRH service utilization

### Measurement and operational definition

**Urban residence**s are characterized by high population density and typically include large cities with significant numbers of inhabitants living in relatively small geographic areas. These areas have greater access to essential services and infrastructure, such as healthcare facilities, schools, electricity, clean water, and transportation networks. Urban regions often feature well-developed road systems, public transportation, and communication networks. There is also a higher prevalence of businesses, offices, factories, and commercial establishments. Urban areas are often officially designated by the government and include regional capitals, major cities, and towns recognized for their administrative, commercial, or industrial importance (34).

**Rural residences** are characterized by low population density, encompassing villages and small settlements where inhabitants are spread out over larger geographic areas. Residents in rural areas typically have limited access to essential services and infrastructure. Healthcare facilities, schools, electricity, and clean water supply may be scarce or of lower quality compared to urban areas. Additionally, transportation networks are less developed, and public services are often less accessible. The economy in rural areas is predominantly agricultural, with most residents engaged in farming, livestock rearing, and related activities. Rural areas are often managed at the local or village level and may include traditional and community-based structures (34).

**Sexual and reproductive health services utilization** refers to the utilization of the essential service package such as modern contraceptive services, HIV testing and counseling (VCT) services, maternal and newborn care services, safe abortion and post-abortion care services, and STI diagnosis and treatment services within the last 12 months. This essential service package is based on the Nairobi Summit Recommendation (ICPD + 25) on essential packages of SRH services for adolescents using the life course approach (10). Adolescents were asked whether they had used SRH services in the past 12 months. The response was measured on a binary scale as “yes” for those who used the services and “no” for those who did not use the services. To confirm a positive answer (“yes”), adolescents were also asked about the type of SRH services they had used and the names of the facilities they had last visited.

**Parents:** This study refers to all individuals who hold significant influence in an adolescent's life and offer unpaid care, irrespective of being biologically related. This encompasses not only biological parents (both mother and father) but also grandparents, older relatives, and other caregivers who provide support without financial compensation (39).

**Family structures** are about cohabitation with biological parents. The family structure variable is obtained from the questionnaire that assesses the living arrangements of adolescents. Adolescents were asked, “With whom have they been living during the last 12 months?” The responses were re-coded as “1” if they lived with two parents' families, “2” if they lived with single-parent families (with either mother or father), and “3” with neither parent family (21, 40).

**Parent-adolescent communication:** It was assessed the following using eight yes or no questions by asking the adolescents if they discussed SRH components with their parent/guardian. The key SRH components include (condom use, STIs /HIV/AIDS, sexual intercourse, menstruation, unwanted pregnancy, contraception, changes during puberty, and choice of sexual friends) with their parents in the last 12 months (21, 41). The response was coded as “1” if they discussed at least two SRH topics in the previous 12 months (21, 41). In this study, the reported Cronbach's alpha of the whole scale was 0.8628

**Paternal monitoring:** was measured using the following five questions: “How often do their parents know what they do in their free time, where they go in the evenings, who their friends are, how they spend their money, and where they go to school”. These Questions were adapted from the previous study conducted in Ethiopia (42). The response was “not at all” (coded as 1) to “always” (coded as 4). To obtain a total score, each item was summed. The score ranges from 5 to 20. Higher values indicate higher levels of parental monitoring. The overall Cronbach's alpha value reported in this study was 0.9022.

**Perceived Social norm:** It was assessed by using single questions. The adolescent was asked whether adolescents are allowed to access SRH services in the community (43). Response Coded 1 if said “Yes”, 0 if not.

**Knowledge of SRH rights:** was measured using 24 items that assessed participants' understanding of SRH rights with Yes = 1 (correct) or No = 0 (incorrect) responses. The total score was determined by summing all 24 items. Responses were then dichotomized based on the mean score: adolescents scoring equal to or higher than the mean were categorized as having “good knowledge,” while those scoring below were categorized as having “poor knowledge” (44, 45).

**Exposure to social media:** Adolescents were asked about their use of social media (e.g., Facebook, Twitter, WhatsApp, Instagram, and TikTok) in the last 12 months. The information was collected as multiple responses from the participants. Based on these responses, we constructed a binary variable where participants who used any social media platforms were considered to have exposure to social media (coded “1”) and (coded as “0”) otherwise (21).

### Data collection and management

Data were collected by twelve trained health professionals with experience in data collection using KoBoToolbox software were selected as data collectors. They were closely supervised by three supervisors who had greater experience in data collection. When selecting data collectors and supervisors, their ability to communicate in the local language and data collection experiences were used as a criterion. The principal investigator provides a two-day extensive training for supervisors and data collectors. The focus of the training is on administering the questionnaire, maintaining confidentiality and privacy, and neutrality during interviews on sensitive topics. After the two-day training, a pilot study was conducted with 5% (60 adolescents) in Chencha District, Gamo Zone, who was not selected for final data collection. After the pilot study, content, and face validation, necessary changes were made, such as removing confusing and unnecessary questions. The final version of the questionnaire was then uploaded to the KoBoToolbox software.

A structured questionnaire was used to collect the data. This tool (questionnaire) was created after reviewing previous studies (11, 13–15, 26, 42). Some of the questionnaires were adapted from the Global Early Adolescent Interview Surveys (46) and the WHO Illustrative Questionnaire (47). The questionnaire was originally developed in English and translated into the local language (Amharic). The data collection tool was face and content validated by reproductive health experts before actual data collection. Data on adolescents' SRH services, individual characteristics, and family environment variables were collected using an interviewer-administered structured questionnaire. Less sensitive questions were asked before the more sensitive ones as recommended for developing a tool for sexual surveys. Each time we visited a village, we revisited the house to get previously absent adolescents to maximize participation rates. The principal investigator and supervisors oversaw the entire data collection process and checked the data for completeness daily. Before the data collectors send the collected data to the center supervisors check the completeness of the questionnaires. In addition, the principal investigator regularly reviewed the files sent to the center by each data collector.

### Data analysis and management

The collected data were cleaned, processed, and analyzed using statistical software STATA version 14.0. Descriptive statistics, such as frequencies, percentages, means, and standard deviations, were calculated and presented through detailed text narratives, graphs, and tables. The reliability of the measurements was assessed using Cronbach's alpha for each composite variable. A Cronbach's alpha score above 0.70 indicates a high level of internal consistency (48).

The dependent variable in this analysis was SRH services utilization (coded 1 = used SRH services and otherwise 0). Two-level multilevel binary logistic regression models were fitted to evaluate factors linked to SRH services utilization at both individual/family and community levels. Since adolescents/households were nested within *Kebeles*, *and Kebeles* nested within districts. Districts were considered random effects to account for unexplained variability at the community level. To assess variability in SRH services use across communities or districts, random effect measures such as the Intra-class Correlation Coefficient (ICC), Median Odds Ratio (MOR), and Proportional Change in Variance (PCV) were calculated. Four models were fitted during multilevel analysis: Null Model (Model 0): Includes only the outcome variable (SRH services use) without predictor variables, serving as a baseline to quantify variability attributable to clustering at the community level. Model I: Incorporates individual/family-level variables as predictors of SRH services use while accounting for community-level effects. Model II: Includes only community-level variables as predictors of SRH services use. Model III: includes both individual/family and community level variables as predictors of SRH services use.

These models were fitted by a Stata command “logit” to identify predictors with the outcome variable. The best-fitting model was selected using log-likelihood ratio (LLR), Akaike Information Criteria (AIC), and Schwarz's Bayesian Information Criteria (BIC). According to the principle of log-likelihood, the higher the better. According to the principle of information criteria (AIC and BIC), the lower the better (49). The model with the highest log-likelihood and lowest AIC was chosen. Finally, crude odds ratio (COR) and adjusted odds ratio (AOR) with 95% confidence intervals were calculated and reported. Variables with *p*-values below 0.05 in the best-fitted model were declared statistically significant.

We employed latent class analysis methods to identify homogeneous groups of adolescents with shared characteristics. This approach helped us better understand the heterogeneity within the adolescent population. We conducted the analysis in two main steps. First, we performed a latent class analysis (LCA) to identify meaningful latent classes of adolescents based on seven sociodemographic indicators. This statistical method used to uncover underlying subgroups of individuals with shared characteristics that are mutually exclusive and exhaustive. Given that the number of latent classes is unknown *a priori*, we estimated a series of models with two to six latent classes. The appropriate number of classes was determined using the Akaike Information Criterion (AIC) and the Bayes Information Criterion (BIC), where lower values indicate a better model fit.

## Results

### Socio-demographic and economic characteristics of adolescents

Out of 1,181 adolescents, 1,172 took part in the study, resulting in a response rate of 99.24%. Among these adolescents, 497 (42.41%) were aged between 10 and 14 years. The average age of respondents was 15.01 years with a standard deviation of 2.69 years. A majority of the participants (56.31%) were female, and 689 (58.79%) lived in urban areas. Regarding school enrollment, 150 (12.80%) adolescents were not attending school during the study period. Additionally, almost half (49.66%) of the adolescents had good knowledge about SRH rights, and 451 (38.48%) reported ever using social media (refer to Table 1).

**Table 1:** Socio-demographic and economic characteristics of adolescents in Gamo Zone, South Regional State of Ethiopia, 2023.

Variables	Category	Frequency	Percentage
Age (in years)	Late adolescents (15–19 years)	641	56.4
	Early adolescents (10–14 years)	497	42.41
Sex of respondent	Male	512	43.69
	Female	660	56.31
Residence	Urban	689	58.79
	Rural	483	41.21
School enrolment status	School enrolled	1,022	87.20
	Not enrolled in school	150	12.80
Religion of respondent	Orthodox	660	51.19
	Protestant	529	45.14
	Muslim	38	3.24
	Other^a^	5	0.43
Attendance at religious services	At least once a week	905	77.22
	Every day	170	14.51
	At least once a month	82	7.00
	Never	15	1.28
Knowledge about SRH right	Good knowledge	582	49.66
	Poor knowledge	590	50.34
Social media exposure	Yes	451	38.48
	No	721	61.52

^a^ Other (Catholic, Apostolic, Adventists, Joba).

### Family or household characteristics of adolescents

Concerning family structure, approximately 871 (74.32%) of the adolescents resided with both parents, while 128 (10.92%) lived with a single parent, and 173 (14.76%) did not live with their biological parents. The majority, 816 (69.62%), of adolescents did not engage in discussions about SRH issues with their parents. The average perceived parental monitoring score among adolescents was 15.01 (±3.57), and the average perceived family connectedness score was 6.66 (±2.25). In terms of family support, approximately 502 (42.83%), 407 (34.73%), and 263 (22.44%) of adolescents reported high, moderate, and low levels of family support, respectively. Moreover, 912 (80.2%) adolescents expressed the belief that the utilization of SRH services by unmarried adolescents was unacceptable in their community (refer to Table 2).

**Table 2:** Family environment-related characteristics of adolescents in Gamo Zone, South Regional State of Ethiopia, 2023.

Variables	Category	Frequency	Percentage
Family structure	Two-parent families	871	74.32
	One-parent families	128	10.92
	Neither-parent families	173	14.76
Adolescent-to-parental discussions regarding SRH issues	Yes	356	30.38
	No	816	69.62
Perceived parental monitoring	(mean ± SD)	(15.01 ± 3.57)	
Perceived family connectedness	(mean ± SD)	(6.66 ± 2.25)	
Perceived social norm	Yes	246	20.99
	No	926	79.01
Family support	Low support	263	22.44
	Moderate support	407	34.73
	High support	502	42.83
Perceived household economic status	Average	850	72.03
	Poor	221	18.86
	Wealthy	101	8.62
Do you know your mother/caregivers use any form of family planning methods?	Yes I know	421	35.92
	No, I did not know	751	64.08

### Adolescent sexual and reproductive health service utilization

The findings of this study reveal that 198 (16.89%) adolescents (95% CI: 14.8%–19.2%) utilized SRH services within the past 12 months. In terms of gender distribution among SRH service users, more than half (59.59%) were female, and 156 (78.79%) of them were from the older adolescent age group. As for their place of residence, over half (57.58%) of the service users resided in urban areas. Modern contraceptive services (*n* = 133) emerged as the most frequently utilized SRH service type, followed by VCT services (*n* = 98). Conversely, the least utilized SRH service types included STI diagnosis and treatment services (*n* = 29), maternal and newborn care services (*n* = 19), and safe abortion and post-abortion care services (*n* = 13). The maximum and minimum frequency with which adolescents visited health facilities to receive SRH services was six and one time, respectively. In terms of where they obtained information about SRH services, over half (60.40%) of the respondents heard about SRH services from healthcare providers, while 304 (50.58%) heard about the services from their peers. The primary reasons cited by young individuals for not utilizing SRH services included not feeling ill (742 or 76.18%), lack of awareness about SRH services (494 or 50.72%), fear of costs for services (172 or 17.66%), and apprehensions about familial or relational reactions (162 or 16.63%), which were the most commonly mentioned reasons (refer to Table 3).

**Table 3:** Adolescent sexual and reproductive health service utilization in Gamo Zone, South Regional State of Ethiopia, 2023.

Variables	Categories	Frequency	Percentage
Ever heard about SRH services	Yes	601	51.28
	No	571	48.72
Source of information about SRH services (*n* = 601)	Health care provider	363	60.40
	Parents	88	14.64
	Friend/Peer	304	50.58
	Teachers	299	49.75
	Social media	272	45.26
The closest healthcare facilities	Health centres	800	68.26
	Private clinics	183	15.61
	Hospitals	113	9.64
	Health posts	73	6.14
Ever used SRH services in the last 12 months	Yes	198	16.89
	No	974	83.11
Types of SRH services used by adolescents during their last visit (*n* = 198)	Modern family planning services	133	67.17
	VCT service	98	49.49
	STI diagnosis and treatment	29	14.65
	Abortion and post-abortion service	13	6.57
	Maternal and New-born care services	19	9.9
Types of health facilities (*n* = 198)	From public health facilities	151	76.26
	Private health facilities	47	23.74
A need of adolescents for SRH services	I don't need it at all.	319	27.22
	I need it slightly.	349	29.78
	I need it seriously.	504	43.00
Commonly mentioned reasons for not using SRH services (*n* = 974)	Not feeling ill	742	76.18
	Lack of awareness about SRH services	494	50.72
	Fear of costs for services	172	17.66
	Apprehensions about familial or relational reactions	162	16.63

### Factors associated with SRH services use among adolescents

#### Random effects analysis

We conducted two-level mixed-effects multivariable logistic regression that is aimed at identifying individual-level and community-level factors associated with SRH services used by adolescents in Gamo Zone. During the analysis, four separate models were fitted to reach the full model. The first level was the null model (empty model without variables), which was used to test the random effect of between and within-cluster variability which is determined by using intra-cluster correlation (ICC). The higher the ICC, the more relevant the community characteristics for understanding individual variation in the outcome variable. In model 0 (empty model), the ICC indicated that 34.06% of the total variability for SRH services use was due to differences between districts while the remaining unexplained 65.94% of the total variability of SRH services use was attributable to individual differences. The variation between- districts increase to 34.48% in Model I (individual/family level only). The variation between- districts increases to 34.95% at the community level only (Model II), while the ICC increases to 34.69% in the complete model with both the individual/family and community level factors (Model III). A widely used statistic for comparing models in multilevel statistical models is Log-likelihood (Log-LL), Akaike information criteria (AIC), and Bayesian information criteria (BIC). So, the full model (Model IV) had higher Log-likelihood results (−337.255) and a lower score of AIC (AIC = 702.5103). Therefore, the full model (Model III), the complete model with both the selected individual and household/community factors, was chosen for predicting the role of individual and household/community factors.

Furthermore, as shown in the table below, the MOR value was 5.01 in the null model, which indicated that there was variation in SRH use between districts. Those who came from the higher SRH services use districts 5.01 times higher chance of SRH services use as compared to respondents from low SRH services use districts (Table 4).

**Table 4:** Random effect analysis for the assessment of individual and community level factors associated with SRH services use among adolescents in Gamo Zone, South Regional State of Ethiopia 2023.

Random effect	Null model	Model-I	Model-II	Model-III
Intra-class correlation (ICC %)	34.06	34.48	34.95	34.69
Median odds ratio	5.01	6.66	6.76	6.72
Proportional change in variance (PCV %)	Reference	13.72%	10.35%	24.86%
Model comparison statistics				
Log-likelihood	−469.228	−342.977	−424.426	−337.255
Akaike information criterion	942.456	707.954	858.853	702.510
Bayesian information criterion	952.589	763.655	884.186	773.441

#### Fixed effect analysis

After adjusting for individual and community-level factors, adolescents with good knowledge about SRH rights were found to have significantly higher odds of using SRH services than those with poor knowledge (AOR = 4.65: 95% CI: 2.68, 8.07). Conversely, adolescents enrolled in school during the data collection period were 81% (AOR = 0.19; 95% CI: 0.11, 0.33) less likely to use SRH services as compared to their counterparts. Furthermore, adolescents living with a single parent were 4.13 times more likely to use SRH services compared to those living with both biological parents (AOR = 4.13; 95% CI: 2.39, 7.12). Similarly, adolescents who discussed SRH issues with their parents had 3.17 times higher odds of using SRH services as compared to their counterparts(AOR = 3.17; 95% CI: 1.89, 5.29). In terms of family support, adolescents with high family support were nearly twice as likely to use SRH services compared to those with low family support (AOR = 1.96; 95% CI: 1.09, 3.51). Additionally, access to social media significantly influenced the likelihood of SRH services use. Adolescents exposed to social media were almost twice as likely to use SRH services compared to those with no exposure (AOR = 1.98; 95% CI: 1.25, 3.15) (Table 5).

**Table 5:** Multilevel mixed effect analysis of individual and community level factors associated with SRH services use among adolescents in Gamo Zone, South Regional State of Ethiopia 2023.

Variables	Categories	Odds ratio (95% confidence interval)			
		Null model	Model I	Model II	Model III
Age of respondent	Younger adolescents	Ref	Ref		Ref
	Older adolescents		1.23 (0.75, 2.01)		0.94 (0.55, 1.59)
School enrollment status	School enrolled		0.19 (0.12, 0.33)*		0.19 (0.11, 0.33)***
	Not enrolled in school	* *	Ref		Ref
Knowledge about SRH right	Good knowledge		5.33 (3.10, 9.14)*		4.65 (2.68, 8.07)***
	Poor knowledge		Ref		Ref
Family structure	Two-parent families		Ref		Ref
	One-parent families		3.93 (2.29, 6.73)*	* *	4.13 (2.39, 7.12)***
	Neither-parent families		1.39 (0.77, 2.52)		1.50 (0.82, 2.74)
Parent-adolescent communication	Yes		3.85 (2.38, 6.26)*		3.17 (1.89, 5.29)***
	No		Ref		Ref
Family Support	Low support		Ref		Ref
	Moderate support		1.53 (0.87, 2.69)		1.62 (0.90, 2.89)
	High support		1.89 (1.08, 3.32)*		1.96 (1.09, 3.51)***
Parental Monitoring mean (± SD)			0.93 (0.87, 0.99)*	* *	0.94 (0.88, 1.01)
Community level variables					
Residence	Urban			Ref	Ref
	Rural			0.82 (0.46, 1.45)	1.04 (0.53, 2.01)
Perceived social norm	Yes			2.77 (1.84, 4.16)**	1.48 (0.89, 2.49)
	No			Ref	Ref
Social media use	Yes			3.47 (2.39, 5.02)**	1.98 (1.25, 3.15)***
	No			Ref	Ref

Ref, reference category.

* *p* < .05 at Model I (individual/family level only).

** *p* < .05 at Model II (community-level only).

*** *p* < .05 at Model IV (full model).

#### Finding from latent class analysis

In the process of selecting the optimal model for Latent Class Analysis (LCA), we evaluated models with 2–6 latent classes using various statistical criteria: Akaike Information Criterion (AIC), Bayesian Information Criterion (BIC), Adjusted Bayesian Information Criterion (ABIC), and Entropy. The goal was to identify the model that best balances fit and complexity.

For the 2-class model, the AIC was 10,078, BIC was 10,164, ABIC was 10,110, and Entropy was 0.76. The 2-class model exhibited the highest Entropy value, indicating clear class separation; however, it might oversimplify the data, as reflected by its relatively high AIC, BIC, and ABIC values. The 3-class model showed improvement with an AIC of 9,942, BIC of 10,073, ABIC of 9,991, and Entropy of 0.717, suggesting a better fit but with slightly reduced classification clarity compared to the 2-class model.

The 4-class model achieved an AIC of 9,889, BIC of 10,067, ABIC of 9,956, and Entropy of 0.702. This model demonstrated the lowest BIC and ABIC values among all models, indicating a favorable balance between model fit and complexity. Although its Entropy was slightly lower than the simpler models, it still provided a reasonable classification quality. The 5-class model had an AIC of 9,898, BIC of 10,121, ABIC of 9,981, and Entropy of 0.704, showing minimal improvement over the 4-class model in terms of AIC and slightly higher BIC and ABIC, suggesting that the added complexity might not significantly enhance the model's explanatory power.

The 6-class model achieved the lowest AIC value of 9,870, indicating the best fit according to this criterion. However, its BIC was 10,138, ABIC was 9,970, and Entropy was 0.745. While the AIC supports the 6-class model for fit, the higher BIC and ABIC compared to the 4-class model, along with only moderate Entropy, suggest that the increased complexity might not be justified.

Considering all criteria, the 4-class model emerges as the most suitable choice. It offers the best balance between fit and parsimony, as indicated by its lowest BIC and ABIC values, along with reasonably high AIC and acceptable Entropy. Therefore, the 4-class model is used to further analysis, providing a robust and interpretable classification of the data while avoiding unnecessary complexity (Table 6).

**Table 6:** Summary of latent class model selection criteria finding from community based cross-sectional survey in Gamo Zone, South Regional State of Ethiopia.

Class	AIC	BIC	ABIC	Entropy
2	10,078	10,164	10,110	0.76
3	9,942	10,073	9,991	0.717
4	9,889	10,067	9,956	0.702
5	9,898	10,121	9,981	0.704
6	9,870	10,138	9,970	0.745

#### Class profile

Based on seven socio-demographic indicators of SRH service utilization, the four-class model demonstrated optimal fit. The classes were defined as follows: Class 1: “Rural school-enrolled adolescents living with both parents”, Class 2: “Urban school-enrolled adolescents living with both parents”, Class 3: “Vulnerable adolescents with low SRH service utilization”, and Class 4: “Urban disadvantaged female adolescents”.

##### Class 1 (16.19%): rural school-enrolled adolescents living with both parents

**Profile:** This class comprises late adolescents who are enrolled in school, live in rural areas, and reside with both parents. Despite having access to social media, they exhibit poor knowledge about sexual and reproductive health (SRH) rights. The stability of living with both parents and the rural setting are significant features of this group. This group represents a significant minority of the adolescent population, indicating a need for targeted interventions in rural areas to improve SRH knowledge. Programs tailored to leverage social media for SRH education could be particularly effective for this group.

##### Class 2 (33.55%): urban school-enrolled adolescents living with both parents

**Profile:** Class 2 consists of late adolescent females who are currently enrolled in school. They reside in urban areas and have access to social media platforms. Additionally, they possess good knowledge about sexual and reproductive health (SRH) rights. Moreover, they live with both parents, indicating a stable family structure and potentially supportive home environment. This group represents a segment of late adolescent female who are actively engaged in education, have access to information through social media, and benefit from a nurturing family environment. Their urban residence suggests exposure to diverse social opportunities and resources, which may contribute to their overall well-being and development. As they are the second-largest group, these adolescents already possess good SRH knowledge. Efforts should focus on maintaining and enhancing existing education programs.

##### Class 3 (7.16%): urban disadvantaged female adolescent

**Profile:** Class 3 comprises late adolescent females who are not currently enrolled in school. They reside in urban areas and do not have access to social media platforms. Additionally, they lack adequate knowledge about sexual and reproductive health (SRH) rights. Furthermore, they do not live with both parents. This group represents vulnerable segment of late adolescent females who may face barriers to education, lack of access to information about SRH rights, and potentially unstable family environments. This is the smallest group but represents a highly vulnerable population due to their poor SRH knowledge and lack of access to social media. Intensive support services, including alternative education and community-based outreach, are critical for this group to improve their SRH outcomes.

##### Class 4 (43.11%): early adolescents with restricted social media access

**Profile:** This class comprises younger adolescents who are enrolled in school and reside with their biological parents. They do not have access to social media platforms. This group represents a segment of younger individuals who are actively engaged in education but are not connected to social media platforms, possibly due to parental restrictions or personal choice. Their living situation with biological parents implies a stable family environment, which may play a significant role in their upbringing and development. Those are the largest group; interventions targeting this class can have a widespread impact. School-based SRH education programs and early intervention initiatives are essential to improve their knowledge and promote healthy behaviors (Table 7).

**Table 7:** Summary of latent class model class sizes and item response probabilities finding from community based cross-sectional survey in Gamo Zone, South Regional State of Ethiopia.

Latent class	Class 1	Class 2	Class 3	Class 4
Class size Probability	0.1619	0.3355	0.0716	0.4311
Proportion younger adolescents in each class	0.1191	0.064	0.2316	0.8511
Proportion older adolescents in each class	0.881	0.937	0.768	0.149
Proportion of boys in each class	0.497	0.407	0.249	0.469
Proportion of girls in each class	0.503	0.593	0.751	0.531
Proportion of out of school adolescent in each class	0.0468	0.1499	0.8825	0.161
Proportion of school enrolled adolescent in each class	0.953	0.850	0.118	0.984
Proportion of adolescent residing in rural area	0.966	0.162	0.227	0.429
Proportion of adolescent residing in urban area	0.0336	0.8377	0.7731	0.5708
Proportion of adolescents who had poor knowledge about SRH right	0.575	0.154	0.554	0.740
Proportion of adolescents who had good knowledge about SRH right	0.425	0.846	0.446	0.260
Proportion of adolescents who do not have access to social media	0.416	0.239	0.711	0.967
Proportion of adolescents who have access to social media	0.5839	0.7608	0.2893	0.0334
Proportion of adolescents living with both biological parents	0.9025	0.7108	0.0406	0.8214
Proportion of adolescents living with single parents	0.0875	0.1278	0.1354	0.0986
Proportion of adolescents living with neither parent.	0.0100	0.1615	0.8240	0.0800

## Discussion

The purpose of this study was to analyze the individual/family and community-level factors impacting the utilization of SRH services among adolescents in the Gamo Zone, Southern Regional State of Ethiopia. The findings of this study highlighted the multifaceted nature of factors influencing adolescent SRH service utilization. They emphasized the importance of holistic, tailored interventions that address individual, familial, and community-level factors. Key findings underscored in this study were adolescents' knowledge of SRH rights, access to social media, family support, parent-adolescent communication, and school enrollment status. These findings offer valuable insights into avenues for improving SRH outcomes within this demographic, highlighting the interconnected nature of various influences on adolescent SRH behavior.

The findings of this study reveal that SRH services utilization among adolescents in the past 12 months was notably low (16.89%, 95% CI: 14.8%–19.2%). In comparison to previous studies conducted in different settings such as Southern Ethiopia (32.8%) (11), Dire Dawa City (39.5% (50), Eastern Ethiopia (23.5%) (7), Nigeria (23.4%) (22), and Bhaktapur district, Nepal (24.7%) (51), the SRH services utilization observed in our study is lower. Further analysis from our quantitative study indicates that the utilization of SRH services was lower among adolescents residing in rural areas, younger adolescents, and boys compared to those living in urban areas, older adolescents, and girls. This pattern aligns with findings from previous studies (41, 51). The findings underscore significant concerns regarding the low utilization of SRH services among adolescents, suggesting potential gaps in access and provision. Additionally, the comparison with previous studies conducted in various settings reveals a concerning trend of lower SRH services utilization in the current study, emphasizing the necessity of identifying the root cause to design targeted interventions to improve access and utilization, especially in the study area. Moreover, the disparities in SRH services utilization based on factors such as rural residence, age, and gender underscore the need for tailored approaches to address these inequalities. Efforts should focus on overcoming barriers faced by adolescents in rural areas, younger age groups, and boys to ensure inclusivity and equity in service provision. Overall, these findings emphasize the ongoing need for research and action to enhance SRH services utilization among adolescents to ensure universal access to comprehensive SRH care.

Adolescents with good knowledge about SRH rights exhibited significantly higher odds of utilizing SRH services compared to those with poor knowledge. This observation finds support in research conducted across various regions including western Ethiopia, Nigeria, and Indonesia (52–54). This might be because those adolescents with better knowledge about their SRH rights may feel more empowered to seek out and access SRH services. Moreover, their informed understanding of the benefits of utilizing such services and the potential consequences of abstaining from them could also contribute to this trend. These findings underscore the importance of comprehensive SRH education initiatives aimed at enhancing adolescents' knowledge and awareness of their rights. Such programs hold promise in not only facilitating greater access to SRH services but also in fostering improved health outcomes among adolescents. Consequently, there is a pressing need for longitudinal investigations to establish causal relationships, as well as for the exploration of interventions geared towards augmenting SRH knowledge among adolescents. Furthermore, it is imperative to delve into additional factors that influence the utilization of SRH services among this demographic group.

Adolescents with high levels of family support show nearly double the likelihood of utilizing SRH services compared to those with low family support. These findings echo existing research highlighting the pivotal role family support plays in shaping adolescent SRH behaviors (40, 55–57). It suggests that robust family support fosters an environment conducive to open discussions about SRH topics, thereby enhancing awareness and motivation to seek out essential services. This underscores the critical importance of acknowledging the impact of family support on adolescent SRH behaviors. Furthermore, it underscores the urgency of bolstering family support mechanisms to facilitate adolescents' access to SRH services effectively. This could involve initiatives aimed at equipping parents with resources and guidance to support their children's SRH needs adequately. To gain a deeper understanding of the complex dynamics of family support and its influence on adolescent SRH, there is a clear call for further comprehensive mixed-methods research.

In consideration of family structure, results showed that adolescents from single-parent families (either mother or father) were more likely to use SRH services than adolescents living in families with both parents. This is consistent with previous research in central Ethiopia and eastern Ethiopia, which showed that adolescents living with both biological parents are less likely to utilize SRH services (21, 50). This may be because those who live with both biological parents may have high parental control compared to single-parental families (58). These adolescents from single-parent families (either mother or father) may have more freedom to communicate with relatives and friends about SRH issues (23). To gain a deeper understanding of the complexities of family dynamics and their influence on the SRH of adolescents, the findings underscore the importance of employing mixed methods designs that integrate qualitative techniques. Such approaches can provide richer insights into the intricate interplay between family structure and SRH outcomes among adolescents. Moreover, initiatives focused on engaging parents to enhance access and utilization of SRH services must be tailored to the specific context of the target population. Recognizing the diverse familial and cultural backgrounds of adolescents, interventions should be flexible and responsive to the unique needs and preferences of adolescents. Therefore, designing family-based interventions aimed at developing positive family life skills is important to improve adolescents’ uptake of SRH services (59). This family approach is essential for effectively involving parents in promoting adolescent SRH and ensuring the success of SRH programs within communities.

Adolescents who have access to social media are nearly twice as likely to utilize SRH services compared to those without such exposure. These results are consistent with existing literature regarding the impact of social media on adolescent SRH behaviors (60–62). This underscores the significance of acknowledging social media's role in shaping adolescent SRH behaviors (63). This suggests that exposure to SRH-related information and resources on social media platforms may enhance awareness and motivate adolescents to seek out SRH services. Consequently, there is a pressing need to utilize social media platforms as vehicles for promoting SRH awareness and fostering service uptake among adolescents. Potential strategies include implementing targeted informational campaigns and establishing partnerships with influential online influencers (64). These findings underscore the importance of further research and collaborative endeavors to fully harness the potential of social media as a tool for advancing adolescent sexual and reproductive health.

In this study, the open communication between parents and adolescents regarding SRH topics emerged as a significant factor influencing adolescents' utilization of SRH services. Most previous studies consistently underscored the crucial role of parent-adolescent communication in imparting knowledge and sharing experiences concerning SRH issues (21, 41). Parental resistance to discussing sex with adolescents stems from a lack of knowledge and sociocultural norms surrounding sexual communication and fear that communication may promote sexual behavior (65). This creates large gaps in knowledge across multiple generations because experiences are not passed down through the family line (40, 66). To foster responsiveness among adolescents, it is overbearing to embrace change and establish consensus within families regarding the open discussion of SRH issues (67). Thus, bridging the gap within the family on SRH issues will improve the sustainability of positive changes and the transmission of accumulated experiences. Future researchers also need to explore how to bridge the gaps in the family in such culturally sensitive communities.

School enrollment status was statistically significantly associated with adolescents' utilization of SRH services. Those adolescents who were out of school were more likely to use SRH services compared to those adolescents who were enrolled. This finding is supported by prior studies undertaken in eastern Ethiopia, Kenya, and southeastern Nigeria (50, 68, 69). Another study from Ethiopia also shows that adolescents who received SRH information from their school teachers were less likely to use SRH services (20). This may be related to a lack of comprehensive sex education for school-enrolled adolescents and the inconvenience of working hours for school-enrolled adolescents (18). Previous studies also showed that the inconvenience of working hours was the main reason for school adolescents not to use SRH services; they spent their time at school during regular health facility working hours (18, 20). Another study conducted in eastern Ethiopia also shows that adolescents are more likely to use SRH services if they find that the opening times of SRH service facilities are convenient for working hours (50). This finding implies the need to revise SRH services provision time convenient for adolescents.

## Strength and limitation of study

The strength of the study lies in its comprehensive approach to understanding adolescent utilization of SRH services in the Gamo Zone, South Regional State of Ethiopia. Through the utilization of a multilevel mixed effects analysis, the study acknowledges the complex and hierarchical nature of the data, enabling a nuanced examination of both individual and contextual factors influencing service utilization. Furthermore, the incorporation of latent class analysis allows the study to identify distinct subgroups of adolescents based on their patterns of service utilization. This approach provides invaluable insights into the diverse needs and preferences of the target population. By strengthening analytical rigor and enhancing the reliability and validity of the results, the study is well-positioned to inform targeted interventions and policy recommendations aimed at improving access and uptake of services among adolescents.

However, this study has several limitations that should be acknowledged. First, we collected data only from adolescents who participated in the study. The lack of data from their parent's or caregivers' perceptive is another weakness of the study. In addition, this study is a cross-sectional study that does not establish a causal relationship between the dependent variable and the independent variables. There are also many factors this study did not assess, including parental style, parental self-efficacy, parental connectedness, and community influences (neighborhood). Future research may attempt to address these factors into consideration to predict SRH services use among adolescents. In addition, further exploring how to bridge the SRH gaps in the family in such culturally sensitive communities is needed. Another limitation is social desirability bias may occur as responses relate to the use of SRH services they may give inaccurate responses there may be over- or underestimation. To minimize this bias, efforts were made to provide a safe environment like the data collectors and supervisors were young health professionals. Also, clear instruction was given to adolescents about the voluntaries of participation; they have the right to answer all or some of the questions. All personal identifying variables were omitted from the questionnaire.

## Conclusions

In conclusion, our study sheds light on the utilization of ASRH services in the Gamo Zone, revealing that 16.89% of the participants accessed these services within the past year. Our findings underscore the significance of timely access to SRH services in addressing distinct challenges faced by adolescents and promoting positive health outcomes. Significant factors associated with SRH service utilization included good knowledge about SRH rights, belonging to one-parent families, engaging in parental discussions regarding SRH issues, high family support, and enrollment in school. It is noteworthy that access to social media was also linked to increased utilization of SRH services among adolescents, emphasizing the evolving role of technology in influencing health-seeking behaviors. Furthermore, our latent class analysis identified four distinct classes of adolescents based on socio-demographic indicators, highlighting the heterogeneity within this population. These classes encompassed a range of characteristics, from rural school-enrolled adolescents living with both parents to urban disadvantaged female adolescents, illustrating the diverse needs and experiences of adolescents in accessing SRH services. These findings underscore the importance of tailored interventions and targeted approaches to address the multifaceted factors influencing SRH service utilization among adolescents. By understanding the individual, familial and community-level factors associated with SRH service utilization, policymakers and healthcare practitioners can develop comprehensive strategies to promote access and utilization of SRH services among adolescents in the Gamo Zone and similar settings.

## Implications this finding

The implications of this study extend to various stakeholders involved in adolescent SRH promotion and service provision. For policymakers, the findings underscore the importance of investing in comprehensive SRH education initiatives tailored to adolescents. Recognizing the critical role of education in promoting informed decision-making and access to services, policymakers can prioritize funding and support for age-appropriate, culturally sensitive SRH programs implemented across diverse settings. Healthcare providers can utilize the study's insights to enhance their approach to adolescent SRH care. By recognizing the influence of family support, effective communication, and social media on adolescents' SRH behaviors, healthcare providers can adopt more holistic and patient-centered approaches. This may involve facilitating family-based interventions, improving communication skills, and leveraging social media platforms to disseminate accurate SRH information. Overall, the implications of this study underscore the importance of collaborative efforts among policymakers, healthcare providers, educators, and community leaders to promote adolescent SRH and well-being effectively. By addressing the multifaceted factors influencing adolescent SRH services utilization, stakeholders can work towards ensuring equitable access to SRH services and improving SRH service use among adolescents.

### Policy and Practical Implications of finding

The research findings have several implications for existing policies and programs related to adolescent sexual and reproductive health (SRH) in Ethiopia:

1. **Education and Awareness Campaigns:** The significant association between good knowledge about SRH rights and increased SRH service utilization highlights the importance of comprehensive SRH education for adolescents. Existing policies and programs should prioritize the implementation of education and awareness campaigns that provide accurate and age-appropriate information on SRH rights, including access to services and decision-making autonomy.
2. **Parental Involvement in SRH Education:** The research underscores the positive impact of engaging in parental discussions regarding SRH issues on adolescent service utilization. Existing policies and programs should encourage and facilitate open communication between parents and adolescents about SRH topics, emphasizing the importance of parental involvement in SRH education initiatives.
3. **Enhanced Family Support:** High family support was found to be significantly associated with increased SRH service utilization among adolescents. Policymakers should explore strategies to strengthen family support networks, including interventions that promote positive parent-child relationships, foster supportive family environments, and enhance parental guidance and counseling on SRH matters.
4. **School-Based Interventions:** The lower likelihood of SRH service utilization among adolescents enrolled in school highlights the need for targeted interventions within educational settings. Existing school health programs should incorporate comprehensive SRH education and services, including access to counseling, contraceptives, and referral pathways to SRH facilities, to ensure that enrolled adolescents receive adequate support for their SRH needs.
5. **Utilization of Digital Platforms:** The association between access to social media and increased SRH service utilization underscores the potential of digital platforms in promoting adolescent SRH. Policies and programs should leverage social media and other digital technologies to disseminate SRH information, provide online counseling and support services, and facilitate access to SRH facilities for adolescents, especially those with restricted social media access.

The following tailored policy and program interventions are recommended to address the specific needs and circumstances of each class of adolescents:

❖ For Rural School-Enrolled Adolescents Living with Both Parents:
  • Policy Intervention: Strengthening Rural Health Infrastructure
    ▪ Allocate resources to improve access to adolescent center sexual and reproductive health services in rural areas, including the establishment of adolescent clinics or satellite clinics in rural schools.
    ▪ Provide incentives for healthcare professionals to work in rural areas and offer specialized training on adolescent SRH to enhance service provision.
  • Program Intervention: School-Based Outreach Programs
    ▪ Establish school-based outreach programs targeting rural school-enrolled adolescents living with both parents.
    ▪ Implement comprehensive SRH education programs within rural schools, covering topics such as puberty, contraception, and STI prevention.
    ▪ Conduct outreach activities in collaboration with local schools to provide SRH information and services to adolescents in rural communities, including mobile clinics and community health worker visits.
❖ For Urban School-Enrolled Adolescents Also Living with Both Parents:
  • Policy Intervention: Integrating SRH Services into Urban Schools
    ▪ Advocate for policies that mandate the integration of adolescents center SRH services into urban schools, including counseling, contraceptive provision, and STI testing and treatment.
    ▪ Provide funding and technical support to urban schools to establish SRH clinics or designate existing health facilities as adolescent friendly centers.
  • Program Intervention: Peer Education and Support Programs
    ▪ Implement peer education and support programs within urban schools, training older students to serve as peer educators and mentors on SRH topics.
    ▪ Establish support groups for adolescents living with both parents, providing a safe space for discussions on SRH issues, peer support, and access to resources and referrals.
❖ For Urban Disadvantaged Female Adolescents:
  • Policy Intervention: Targeted Support for Vulnerable Populations
    ▪ Develop targeted policies and programs to address the unique needs of urban disadvantaged female adolescents, including those from low-income households or marginalized communities.
    ▪ Allocate funding for community-based organizations and NGOs to implement outreach and support programs tailored to the needs of this population.
  • Program Intervention: Comprehensive SRH Services and Empowerment Programs
    ▪ Deploy community health workers to conduct door-to-door outreach targeting urban disadvantaged female adolescents, offering comprehensive services, including contraception, STI testing and treatment, and gender-based violence support.
    ▪ Provide personalized SRH counseling and support, addressing issues such as family planning, pregnancy prevention, and gender-based violence.
    ▪ Implement empowerment programs focused on building self-esteem, leadership skills, and economic opportunities for disadvantaged female adolescents, enabling them to make informed choices about their SRH and overall well-being.
    ▪ Facilitate access to SRH services through mobile clinics or referral systems, ensuring confidentiality and privacy for adolescents seeking care.
❖ For Early Adolescents with Restricted Social Media Access
  • Policy Intervention: Enhancing Digital Literacy and Access
    ▪ Develop policies to promote digital literacy and expand access to online resources and information for early adolescents with restricted social media access.
    ▪ Invest in initiatives to bridge the digital divide, including providing subsidized or free internet access and digital devices to underserved communities.
  • Program Intervention: Peer Education and Support Groups
    ▪ Establish peer support networks for early adolescents with restricted social media access.
    ▪ Train peer support networks to deliver age-appropriate SRH information and facilitate discussions on topics such as puberty, body image, and healthy relationships.

## Data Availability

The original contributions presented in the study are included in the article/Supplementary Material, further inquiries can be directed to the corresponding author.
